# A Minimally Invasive Fixation Versus Double Plating of Associated Posterior Malleolus and Fibula Fractures—A Comparative Human Cadaveric Biomechanical Study

**DOI:** 10.3390/medicina61101847

**Published:** 2025-10-15

**Authors:** Konstantin Ganchev, Preslav Penev, Ivan Zderic, Kajetan Klos, R. Geoff Richards, Dimitar Raykov, Boyko Gueorguiev, Lionel Llano, Karl Stoffel

**Affiliations:** 1 AO Research Institute Davos, 7270 Davos, Switzerland; ko.ganchev@gmail.com (K.G.); ivan.zderic@aofoundation.org (I.Z.); geoff.richards@aofoundation.org (R.G.R.); lionel.llano@hospitalitaliano.org.ar (L.L.); 2 Faculty of Medicine, Medical University “Prof. Dr. Paraskev Stoyanov”–Varna, 9002 Varna, Bulgaria; dr_penev@abv.bg (P.P.); kajetan.klos@freenet.de (K.K.); raikovortho@dir.bg (D.R.); 3 Meliva Gelenkzentrum, 55116 Mainz, Germany; 4 Department of Trauma, Hand, and Reconstructive Surgery, University Hospital Jena, 07747 Jena, Germany; 5 Hospital Italiano de Buenos Aires, Buenos Aires C1199, Argentina; 6 University Hospital Basel, University Basel, 4031 Basel, Switzerland; nkstoffel@hotmail.com

**Keywords:** ankle fracture, plate osteosynthesis, fibula nail, intramedullary nail, motion tracking, posterior malleolus

## Abstract

*Background and Objectives:* Ankle fractures are common and occur in up to 25% of cases with posterior malleolus (PM) involvement. The gold standard for their treatment considers posterior approaches and plating of both the PM and fibula. However, in elderly and comorbid patients, this strategy remains controversial. The objective of this biomechanical study was to compare a minimally invasive fixation—utilizing a fibula nail and percutaneous anteroposterior (AP) screws—versus double plating. *Materials and Methods:* An oblique fibula fracture associated with a Haraguchi type 1 PM fracture was reproduced in sixteen human cadaveric specimens randomized to two groups. Eight specimens were treated with a fibula nail plus two AP screws fixing the PM, while the remaining eight specimens underwent double plating. Biomechanical testing was performed under destructive complex cyclic loading applying a staircase protocol. Interfragmentary movements were captured via motion tracking. *Results:* Initial axial stiffness was similar between nailing (1125.9 ± 341.7 N/mm) and double plating (742.9 ± 600.1 N/mm) (*p* = 0.129). During cyclic testing, interfragmentary fibula displacement was higher for double plating versus nailing (*p* = 0.057), whereas PM displacement and syndesmosis diastasis remained comparable between the two techniques (*p* ≥ 0.197). *Conclusions:* The minimally invasive fixation of associated PM and fibula fractures utilizing a fibula nail and two anteroposterior screws demonstrated non-inferiority to double plating and presents a viable option in cases where delicate soft tissue management is required.

## 1. Introduction

Ankle fractures account for up to 10% of all adult fractures, with an increasing incidence, especially in elderly patients [1,2]. Moreover, the posterior malleolus (PM) is fractured in up to 25% of cases and requires surgical fixation [3]. As the population continues to age and life expectancy increases, concerns have been raised about treatment outcomes in the presence of significant multiple comorbidities and limited compliance. The reported wound complication rate in different patient groups reaches up to 20% [4,5,6,7].

Plating is a well-established standard for the surgical treatment of fibula fractures. Direct visualization through the posterolateral approach (PLA) is the standard for addressing complex fracture patterns with concomitant posterior and lateral malleoli involvement [8]. However, despite the advantages of PLA, the reported early complication rate is high [9].

Morbid patients, unsuitable for the prone position during operation or with disturbed local soft tissue status, represent a challenge for clinicians. The individual patient-based approach has led to the development of minimally invasive strategies to address unstable ankle fractures and vulnerable soft tissue terrain [10,11]. Studies in diabetic and elderly patients demonstrate that intramedullary (IM) nailing of fibula fractures results in lower wound complication rates and less hardware-related pain, allowing for earlier weight-bearing while maintaining stability comparable to plate fixation [12,13,14,15,16,17,18]. On the other hand, a soft tissue-sparing alternative to the direct fixation of the posterior malleolus fragment (PMF) is the percutaneous anteroposterior (AP) screw fixation, which, although considered with inferior stability to buttress plating, provides satisfactory clinical results [19].

New minimally invasive techniques have emerged to address such patients with ankle fractures, and one of them employs the use of a fibular nail and AP screws for PMF fixation. However, the stability of such bone-implant constructs continues to be a concern in clinical practice.

To date, no biomechanical analysis has investigated the fibula nail performance in complex fracture patterns involving a PMF. Therefore, the present study was designed with the aim to (1) investigate whether the minimally invasive osteosynthesis construct implementing an IM interlocking fibula nail and AP screws is biomechanically appropriate for fixation of associated PM and fibula fractures, and (2) compare its biomechanical performance to a plated construct addressing the same fracture pattern. It was hypothesized that the biomechanical competence of both constructs would be comparable.

## 2. Materials and Methods

### 2.1. Specimens and Preparation

In total, 8 right and 8 left fresh-frozen (−20 °C) below-knee human cadaveric specimens from 5 female and 7 male donors aged 75 ± 11 years (mean ± standard deviation, SD) (range 61–90 years) with no history of previous pathology were used. This study was approved by the institutional internal review board based on the approval of the specimen delivery by the Science Care Ethics Committee. All donors gave their informed consent inherent within the donation of an anatomical gift statement during their lifetime.

Bone mineral density (BMD) was evaluated in the calcaneus via computed tomography (CT) scanning (Revolution EVO, GE Medical Systems AG, Opfikon, Switzerland) at a slice thickness of 0.63 mm using a calibration phantom (European Forearm Phantom QRM-BDC/6, QRM GmbH, Möhrendorf, Germany).

Based on BMD, the specimens were randomized to 2 groups for treatment via either fibula nail plus two AP screws for the PM (Group 1) or using double plating (Group 2). The randomization was performed by initial stratification on the basis of BMD and then by random assignment of the treatments within each stratum. A sample size of *n* = 8 was chosen based on a priori power analysis to achieve 80% power at a 5% level of significance, considering two independent-sample groups and a two-sided statistical evaluation under the assumption that the standard deviation in each group is not larger than 60% of the difference in mean values between the groups.

All specimens were defrosted at room temperature for 24 h prior to preparation, instrumentation, and biomechanical testing. An amputation was performed through the proximal one-third of the calf and the Chopart joint in the distal part. The specimens were dissected of soft tissues, including skin, subcutaneous tissue, muscles, and tendons, such that only bones, ligaments, and joint capsules were preserved. Subsequently, the proximal tibia–fibula complex was embedded in a 65 mm long cylindrical form of 48 mm diameter using polymethylmethacrylate (PMMA, SCS-Beracryl, Suter Kunststoffe AG, Jegenstorf, Switzerland). Similarly, the hindfoot was embedded to the level of the subtalar joint in a cylindrical form of 105 mm diameter.

**Figure 1:**
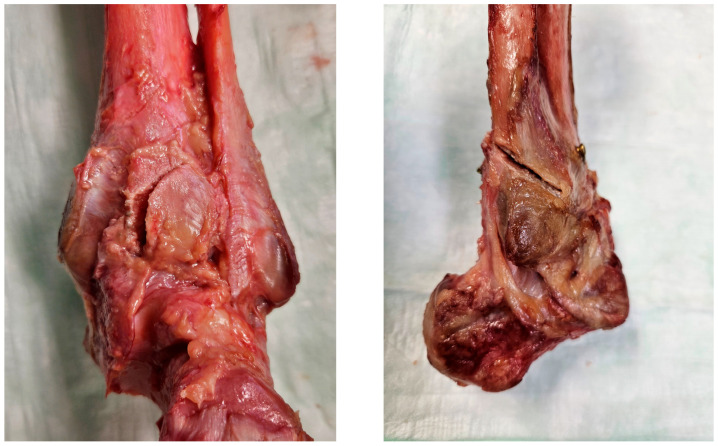
Exemplified specimen in (**left**) posterior view with visualized Haraguchi type 1 posterior malleolus fracture and (**right**) lateral view visualizing Weber type B oblique fibula fracture.

Fracture model creation commenced with an osteotomy of the distal fibula, starting at the anterior margin of the incisura and proceeding in a proximal and posterior direction at a 45° angle, recreating a Weber type B fracture. A posterolateral oblique fracture, Haraguchi type 1, was simulated in the PM with a 30° inclination of the blade, starting the cut 3 cm proximally to its posterior margin. Care was taken to preserve the integrity of the posterior inferior tibiofibular ligament attachment to the PM (Figure 1). The medial malleolus and deltoid ligament were left intact to simulate a successful medial malleolar fixation as previously described [14]. Whereas the posterior inferior tibiofibular ligament was left attached to the PM, the anterior inferior tibiofibular ligament was cut midway with a blade.

In Group 1, the PM was fixed with two 3.5 mm fully threaded cortical lag screws (Johnson & Johnson MedTech, Zuchwil, Switzerland) under direct visualization, placed in AP direction. A ViTUS-Fi Fibula Nailing System (Dieter Marquardt Medizintechnik GmbH, Spaichingen, Germany) was used for nailing of the fibula fracture. A 3.6 mm (diameter), 110 mm long nail was selected according to the surgical technique recommended by the manufacturer. Two monocortical screws were used for the infrasyndesmotic AP interlocking, while the lateral interlocking was performed with one bicortical screw at the level of the syndesmosis and one tricortical screw above this level.

**Figure 2:**
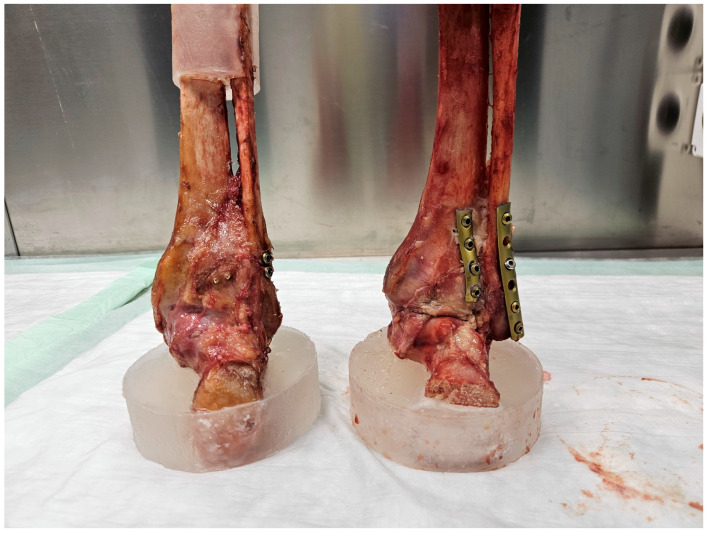
Photographs depicting the posterior side of instrumented specimens of Group 1 (**left**) and Group 2 (**right**) prepared for biomechanical testing. Left: the threads of the two AP screws protruding from the posterior malleolus fragment are visible, together with the lateral interlocking screws of the fibula nail; right: posterior double plating of both the posterior malleolus and fibula fractures.

In Group 2, partial posterior capsulotomy was performed to verify the anatomical repositioning of the PM, which was eventually fixed with a 3.5 mm four-hole buttress plate (1/3 tubular plate, Johnson & Johnson MedTech, Zuchwil, Switzerland) by placing two fully threaded 3.5 cortical screws in the proximal fragment and two interfragmentary fully threaded 3.5 mm cortical screws in the distal fragment perpendicular to the fracture surface. The fibula fracture was fixed according to the corresponding antiglide plate technique, securing either a 5-hole or 6-hole 3.5 mm one-third tubular plate positioned posteriorly on the fibula with four 3.5 mm cortical screws (Johnson & Johnson Medtech, Zuchwil, Switzerland). Two screws were placed in the proximal fragment. One of the two screws in the distal fragment was inserted as a lag screw crossing perpendicularly the fibula fracture line (Figure 2).

**Figure 3:**
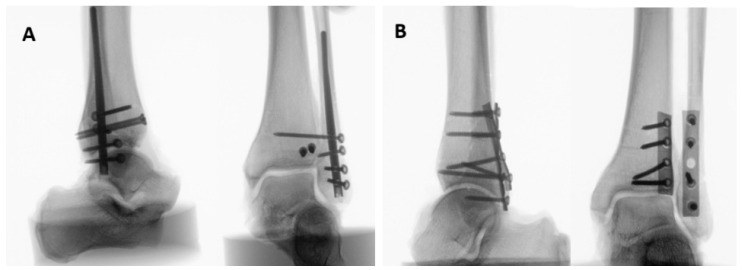
Lateral and AP X-rays of specimens instrumented with either a fibula nail and two AP screws (**A**, Group 1) or with two plates (**B**, Group 2).

Lateral, AP, and mortise X-rays of the final bone-implant construct were shot for each specimen to verify the implants’ position (Figure 3).

To ensure equal quality of fixation in the two groups, their instrumentation was performed by two surgically experienced authors—one per group. Marker sets were attached to the tibia, proximal fibula fragment, distal fibula fragment, and the PM (Volkmann) fragment for motion tracking.

### 2.2. Biomechanical Testing

**Figure 4:**
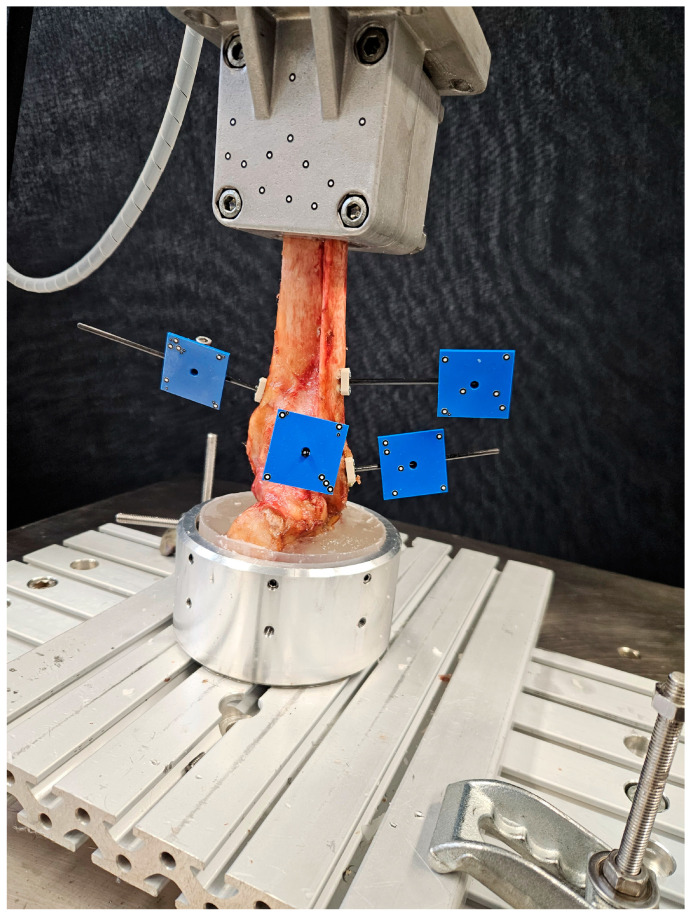
Setup with a specimen mounted for biomechanical testing. Marker sets are attached to the tibia, proximal fibula fragment, distal fibula fragment, and the PM fragment for motion tracking.

Biomechanical testing was performed on a biaxial servohydraulic material testing machine (Bionix 858.20, MTS Systems Corp., Eden Prairie, MN, USA) equipped with a 5 kN/50 Nm load cell (MCS-10, HBK, Darmstadt, Germany). The proximal and distal PMMA-embedded specimen parts were attached via an interconnected cardan joint to the machine transducer and fixed to the machine base by custom holders. The tibia shaft was aligned to the machine axis, while the hindfoot was parallel to the base, simulating weight-bearing in a neutral mid-stance position of the ankle joint (Figure 4).

The loading protocol commenced with a non-destructive quasi-static ramp from 20 N preload to 150 N axial compression at 13 N/s. Then, destructive complex sinusoidal, cyclic, axial and torsional loading was applied at 2 Hz over 17,000 cycles. The specimens were loaded in compression along the machine axis, applying a synchronous torsional loading simulating external rotation of the foot during the stance phase of a walking cycle [20]. The magnitude of cyclic axial loading, starting at 150 N peak compression, was increased in a staircase manner every 1000 cycles by 150 N until the end of the cyclic test. Peak external rotation, starting at 1°, was incrementally increased by 1° every 500 cycles until reaching a peak of 5°, which was then held constant until the end of the cyclic test. The valley axial load was held constant at 20 N, while the valley torsion was constantly held at 0°.

### 2.3. Data Acquisition and Evaluation

Machine data in terms of axial displacement, axial force, angular displacement, and torque were continuously recorded from the machine transducers at 128 Hz throughout the test. Initial axial stiffness was calculated as the slope of the load–displacement curve derived from the initial quasi-static ramp within the linear range of 20–150 N. The three-dimensional coordinates of the attached markers were captured by a stereographic camera system (Aramis SRX; Carl Zeiss GOM Metrology GmbH, Braunschweig, Germany) for motion tracking. The magnitudes of interfragmentary displacement between the tibia and the Volkmann fragment at the most inferior-medial margin, between the proximal and distal fibula fragments at the most anterior-caudal margin, and in the syndesmosis between the distal fibula fragment and the Volkmann fragment at the level of the fibula fracture line were evaluated at the initial stage after the third loading cycle and then intermittently every 1000 cycles until 8000 cycles with respect to the beginning of the cyclic test under peak axial loading condition to evaluate the degradation of the construct stability over the course of cycles. The latest evaluated time point represented the biggest rounded cyclic number with none of the specimens dropping out. The locations of the aspects of interest were virtually registered prior to test start using a touch probe. Furthermore, a margin of 2 mm interfragmentary fibula displacement was set as a criterion for clinically relevant failure, and the number of cycles until fulfillment of this criterion under peak axial loading—defined as cycles to failure—was calculated.

### 2.4. Statistical Analysis

Statistical analysis was carried out using the SPSS software package (V. 27, IBM, Armonk, NY, USA). Evaluation and verification of the normal data distribution were conducted by utilizing Shapiro–Wilk test. To identify significant distinctions between the study groups, Independent-Samples *t*-test was used, whereas progression over time was screened with General Linear Model Repeated Measures test. Level of significance was set to 0.05 for all statistical tests.

## 3. Results

Each group consisted of equal numbers of right and left specimens with BMD of 112.7 ± 36.0 mgHA/cm^3^ in Group 1 and 130.7 ± 47.3 mgHA/cm^3^ in Group 2, with homogeneous distribution between them (*p* = 0.475).

### 3.1. Initial Axial Stiffness

The initial axial stiffness was 1125.9 ± 341.7 N/mm in Group 1 and 742.9 ± 600.1 N/mm in Group 2, with no significant differences between the two groups (*p* = 0.129).

### 3.2. Fracture Displacement

**Figure 5:**
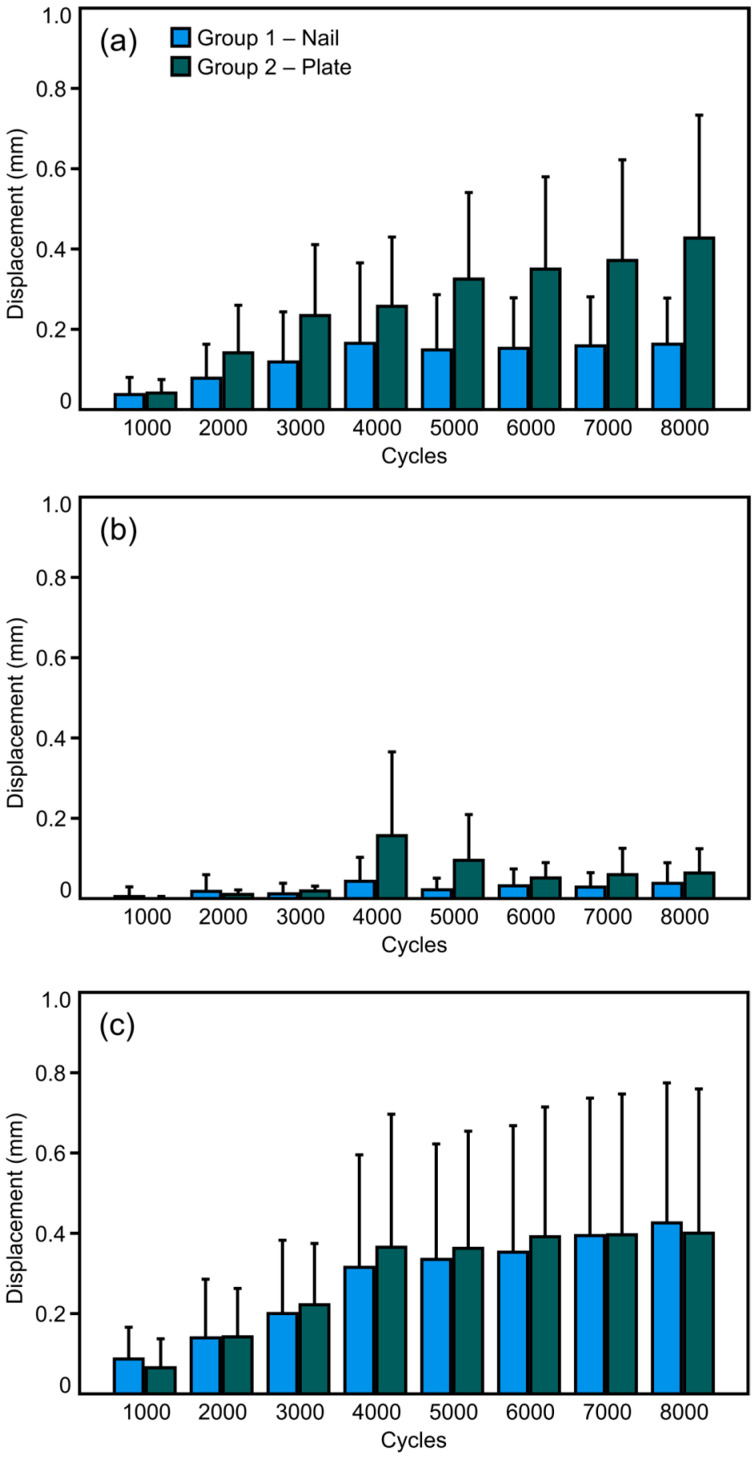
Interfragmentary fibula displacement (**a**), Volkmann fragment displacement (**b**), and syndesmosis diastasis (**c**) over the first 8000 cycles presented in terms of mean value and standard deviation for each separate group (Group 1—nail and Group 2—plate).

The magnitude values of the three investigated parameters—interfragmentary fibula displacement, syndesmosis diastasis, and Volkmann fragment displacement—at the eight intermittent time points over the course of the first 8000 cycles of cyclic testing are displayed in Figure 5. The interfragmentary fibula displacement and the syndesmosis diastasis were associated with a significant increase over time within each group (*p* ≤ 0.041), while the Volkmann fragment displacement remained without a significant change in both groups (*p* ≥ 0.189). The differences between the groups were associated with a trend to significantly higher displacement values in Group 2 versus Group 1 for interfragmentary fibula displacement (*p* = 0.057) and no significant differences between the two groups with regard to both syndesmosis diastasis and Volkmann fragment displacement (*p* ≥ 0.197).

### 3.3. Cycles to Failure

The number of cycles to failure was 13,951 ± 3588 in Group 1 and 14,846 ± 3078 in Group 2, with no significant difference between the two groups (*p* = 0.601).

## 4. Discussion

In the last decade, the interest in fibula nailing has grown tremendously. A clinical meta-analysis from 2022 comparing open reduction and internal fixation (ORIF) versus IM nailing reported less short-term complication rates for the latter while maintaining similar long-term functional outcomes [21]. In a systematic review conducted by Attia et al., IM fibula nailing of ankle fractures resulted in superior 12-month functional results compared to plating [16]. It seems that the current trend increasingly favors the implementation of this technique in the armamentarium of orthopaedic trauma surgeons. Traditionally, the standard for treatment of unstable ankle fractures during the last 60 years has been plate fixation. Any alternative to that long-lasting method should first prove that it is biomechanically adequate in the variety of clinical conditions it might encounter. The most important finding of the current study is the reported biomechanical non-inferiority performance of an alternative minimally invasive technique in comparison to the standard plating of such fractures.

To our knowledge, only four biomechanical studies have been conducted addressing fibula IM fixation so far [12,13,14,15]. Moreover, each of them focused on comparing fibula nailing to plate fixation exclusively for fibula fractures. However, up to 25% of clinical cases of ankle fractures involve a PM fragment, which compromises bony congruency and disturbs syndesmotic stability [1].

Even to date, PM fragment fixation continues to be a controversial topic. The understanding that the PM fixation is necessary only if it involves more than a quarter of the articular surface is considered outdated. Ensuring a stable and precise reduction in the PM is crucial, as anatomical reduction in the notch restores syndesmosis stability and improves both pressure distribution and neutralization of rotational forces [22]. In addition, a stable PM fixation eliminates the need for syndesmotic screw fixation. Despite several biomechanical studies claiming that buttress plating provides the most stable osteosynthesis of the PMF, the clinical results after AP screw fixation present satisfactory functional outcomes with a similar rate of complications and range of dorsiflexion [23,24,25]. The results from the current study confirm that both the syndesmosis diastasis and PMF stability remain comparable between the two fixation techniques.

In 2022, Wordie et al. reported 32 cases of good functional outcome after IM fibula nailing associated with two-AP-screw fixation of the Volkmann fragment in complex ankle fractures in morbid patients whose condition did not allow either prone position during operation or ORIF [11]. Such minimally invasive constructs appear to appreciate the severity of the general condition and the local soft tissues in high-risk patients. However, although a possible routine clinical application of such methods is anticipated, no information about their stability exists yet, which was the reason to perform the current study. In the absence of a biological response, our results confirm that the ankle would withstand weight-bearing in neutral position. However, a generalization of the current findings and their direct translation to the functional outcomes in complex clinical cases would be very difficult from a specific biomechanical setting.

The fracture pattern selection in the present work was based on an epidemiological analysis of ankle fractures in adults. With 63.6% incidence, Weber B is the most frequent fibula fracture type, along with the most common Haraguchi type 1 PM fractures [1,3]. In the present work, the simulated Weber type B fracture was short oblique, as suggested by the Lauge–Hansen classification in the case of supination–external-rotation injuries. With this being said, oblique fibula fractures represent a small portion of the diversity of fractures in this anatomical region. Further studies should be setup to analyze how constructs with comminuted, transverse or long oblique fractures respond to the current testing environment. Similar consideration should be given to PM fractures. Although Haraguchi type 1 is the most common type, no additional comminution, such as by implementing intercalary fragments, was considered in the current investigation. In a clinical situation, a PM comminution would have resulted in additional instability of the whole construct.

In the current study, biomechanical testing was conducted solely in neutral position of the ankle, considering that such patients are immobilized in a walking boot during the early postoperative stage. External rotational forces were applied to the ankle joint to mimic the physiological application of forces in the ankle joint in foot-flat position [26]. Stepwise applied load of 150 N up to 1500 N was considered to simulate the gradual progress from touch weight-bearing to partial weight-bearing and full weight-bearing.

The results of the current study verified that such complex osteosynthesis provides stability within the clinically acceptable boundaries for uneventful bone healing. Although numerous studies show that percutaneous AP fixation provides lesser stability compared to plating of the PM, in the present study, after reaching the maximum of 5° in external rotation, a tendency for greater displacement of the PM fragment was observed after double plating, probably caused by the lack of syndesmotic screw fixation through the fibula. However, trans-syndesmotic fixation was not required for this technique, as the anatomical PM reduction restores the physiological position and movement in the syndesmosis [27]. In contrast, as recommended by Bugler and Backer, a syndesmotic screw was placed during the nailing technique application to provide maximum stability of the fibula fracture [26,28]. As the load increased, the fracture displacement progression was saturated, similarly to the findings of Kohler et al. [12]. In contrast, a linearly increasing displacement was observed after plate fixation [12]. The difference is likely due to the nature of IM nailing, which mainly serves as a weight-bearing fixation. In the absence of proximal interlocking in the fibula fragment, a greater compression is applied at the fracture line under axial loading, similar to the effect of dynamization of femoral or tibia nails. No pathological syndesmotic widening was identified in our cases, as all measured displacement values were within the physiological limit for syndesmotic stability. Such results are expected, as the stress distribution in neutral position of the ankle is spread in a manner that does not greatly affect the syndesmosis.

The current study has several limitations. First, the deltoid ligament was preserved, thus maintaining a portion of the inherent ankle stability. The reason for excluding such damage is that it occurs rarely in advanced-age patients with osteoporosis and local tissue issues and is therefore not of primary concern. Second, a small sample size and lack of biologic response are well-known limitations of human cadaveric studies. Third, the current tests were performed solely in neutral position based on the consideration that the patients should be immobilized after such treatment. However, the biomechanics of the ankle joint is more complex compared to the reproduced simplified testing. Moreover, although the choice of fracture model was based on epidemiological data, its pattern still represents a small portion of the variety of injuries affecting the ankle. That is why this investigation can be considered successful from a single perspective; however, a generalization of the current findings towards all unstable ankle fractures with different patterns would not be justified. Finally, in minimally invasive techniques, perfect anatomical reduction is rarely achievable in clinical conditions, whereas in the present work the reduction was performed under direct visualization.

## 5. Conclusions

From a biomechanical perspective, the minimally invasive fixation of associated posterior malleolus and fibula fractures utilizing a fibula nail and anteroposterior screws demonstrated non-inferiority to the traditional double plating technique and presents a viable option, particularly in cases where delicate soft tissue management is required. Even though precise motion tracking and realistic clinical conditions were biomechanically simulated, further clinical studies are necessary to verify the effectiveness of this minimally invasive technique.

## Data Availability

The datasets analyzed during the current study are available from the corresponding author on reasonable request.
